# The Role of Prebiotics and Probiotics in Prevention of Allergic Diseases in Infants

**DOI:** 10.3389/fped.2020.583946

**Published:** 2020-12-22

**Authors:** Simona Sestito, Enza D'Auria, Maria Elisabetta Baldassarre, Silvia Salvatore, Valeria Tallarico, Ettore Stefanelli, Flora Tarsitano, Daniela Concolino, Licia Pensabene

**Affiliations:** ^1^Pediatric Unit, Department of Medical and Surgical Sciences, University “Magna Graecia” of Catanzaro, Catanzaro, Italy; ^2^Department of Pediatrics, Vittore Buzzi Children's Hospital-University of Milan, Milan, Italy; ^3^Neonatology and Neonatal Intensive Care Unit, Department of Biomedical Science and Human Oncology, “Aldo Moro” University of Bari, Bari, Italy; ^4^Department of Pediatrics, Ospedale “F. Del Ponte”, University of Insubria, Varese, Italy; ^5^Department of Health Sciences, School of Medicine and Surgery, University Magna Graecia of Catanzaro, Catanzaro, Italy

**Keywords:** prebiotics, probiotics, prevention, atopic dermatitis, eczema, synbiotics, allergic diseases

## Abstract

Allergic diseases have been linked to genetic and/or environmental factors, such as antibiotic use, westernized high fat and low fiber diet, which lead to early intestinal dysbiosis, and account for the rise in allergy prevalence, especially in western countries. Allergic diseases have shown reduced microbial diversity, including fewer lactobacilli and bifidobacteria, within the neonatal microbiota, before the onset of atopic diseases. Raised interest in microbiota manipulating strategies to restore the microbial balance for atopic disease prevention, through prebiotics, probiotics, or synbiotics supplementation, has been reported. We reviewed and discussed the role of prebiotics and/or probiotics supplementation for allergy prevention in infants. We searched PubMed and the Cochrane Database using keywords relating to “allergy” OR “allergic disorders,” “prevention” AND “prebiotics” OR “probiotics” OR “synbiotics.” We limited our evaluation to papers of English language including children aged 0–2 years old. Different products or strains used, different period of intervention, duration of supplementation, has hampered the draw of definitive conclusions on the clinical impact of probiotics and/or prebiotics for prevention of allergic diseases in infants, except for atopic dermatitis in infants at high-risk. This preventive effect on eczema in high-risk infants is supported by clear evidence for probiotics but only moderate evidence for prebiotic supplementation. However, the optimal prebiotic or strain of probiotic, dose, duration, and timing of intervention remains uncertain. Particularly, a combined pre- and post-natal intervention appeared of stronger benefit, although the definition of the optimal intervention starting time during gestation, the timing, and duration in the post-natal period, as well as the best target population, are still an unmet need.

## Introduction

**Figure 1:**
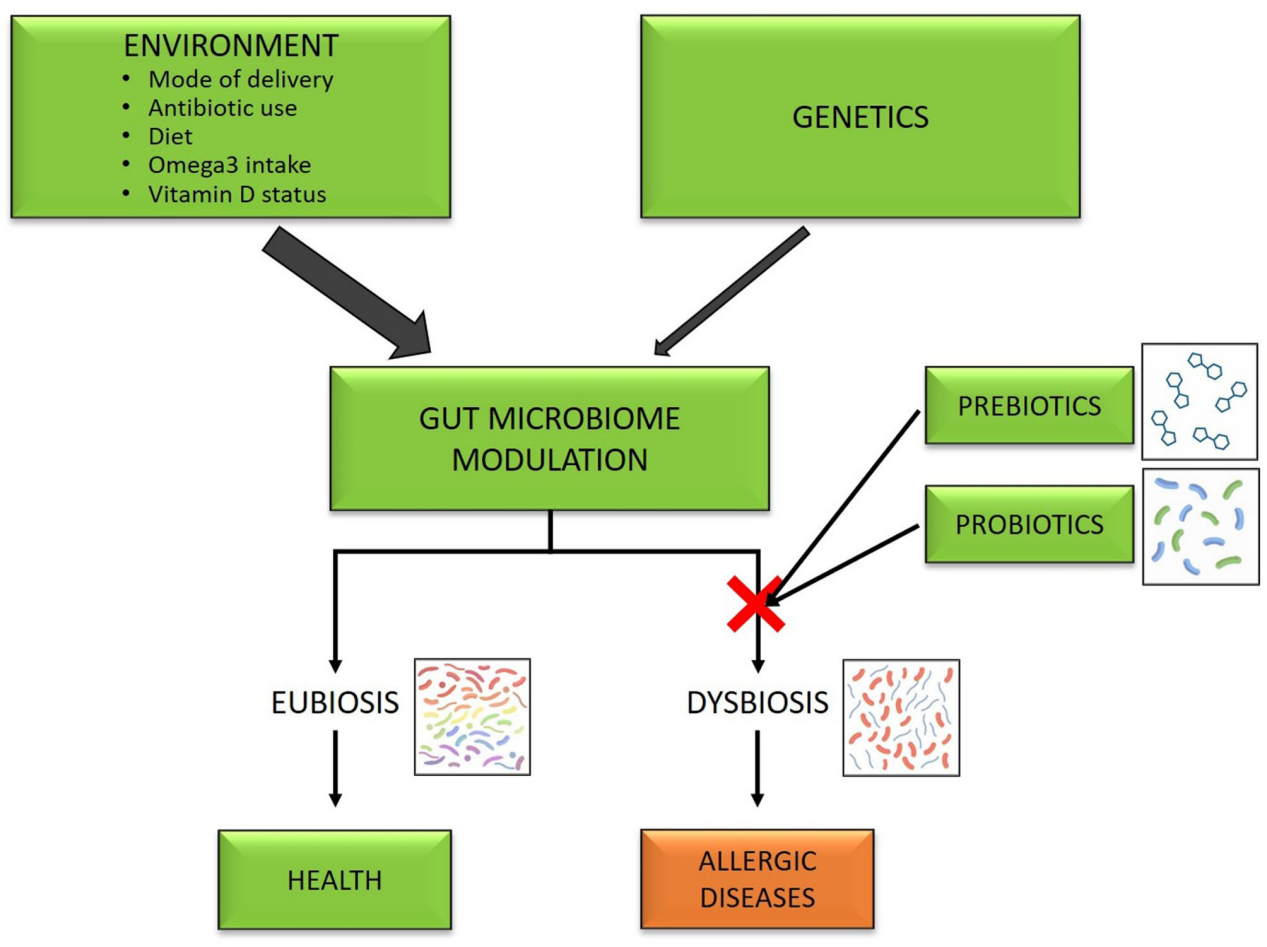
Gut microbiota as a target of prevention of allergic diseases.

Allergic diseases represent a medical challenge and a worldwide burden, in particular in the most developed countries, where the frequency of affected subjects overcome 30% and is still growing (1, 2). Allergic diseases are the result of a complex interaction genome-environment which leads to an alteration of the immune system (3, 4). A lot of genes, HLA, and specific genes identified by genome-wide association studies, have been identified for asthma (5, 6), food allergy (7), and atopic dermatitis (8, 9). In infants, allergic disease prevalence has been associated with the allergic status of the parents, being ~10% in those with a negative family history of atopic disorders and 20–30% in those with allergy in their first-degree relatives (10). Although genetic factors can affect the tendency to the development of allergic diseases, the rapid rise of allergic diseases in the last two decades can be explained by environmental factors (11). A lot of factors related to the environment have been called in cause to explain the rise, especially in western countries. These include mode of delivery, with cesarean delivery representing a risk for atopy, food allergy and asthma (12), antibiotic use (13), westernized high fat and low fiber diet (14, 15), reduction of omega-3-polyunsaturated fatty acids and vitamin D insufficiency or deficiency (16). All the above act on microbiota (Figure 1), which an increasing body of evidence suggests to play a central role in shaping the normal development, and maturation of the immune system (17). Some of the effects on immune programming are thought to be due to epigenetic effects on the expression of genes (18, 19).

In a healthy state, the gut microbiota is in eubiotic status; in contrast, gut dysbiosis, an imbalance in the composition and/or function of the gut microbiota, has been associated with allergic diseases, such as eczema, asthma, and food allergy (20–23). Animal and human studies have found that subjects with allergic disease are carriers of reduced microbial diversity and different proportions of certain microbial species (24).

The establishment of an altered gut microbiota seems to occur in the early stage of development, as demonstrated by studies that have shown that atopic infants vs. non-atopic infants at 1 year of age had different gut composition at 3 weeks of age and 3 months (25). These differences, with allergic diseases showing reduced microbial diversity, including fewer lactobacilli and bifidobacteria, were observed before the onset of clinical symptoms, supporting their possible causative role in allergic diseases (24, 26–28). In agreement with these observations, more recently West et al. (29) reported that the development of atopic eczema is influenced by lack of immune system modulation after birth, mediated by the gut microbiota. The majority of molecular data suggest that gut colonization occurs through contamination shortly after delivery (30, 31) although some recent experiments suggest that it might take places already *in utero* and then further shaped post-natally (32). Therefore, it has been speculated that the recent increase in the prevalence of allergy may be consequent to early intestinal dysbiosis (33). The above hypothesis and observations aroused the interest of research for shaping gut microbiota in the early stages to prevent the development of allergic diseases. Different strategies have been studied, including probiotic, prebiotic, synbiotic supplementation pre-natally, post-natally, or both (34).

Probiotics are “living microorganisms that, at certain doses, may provide health benefits” (10). Probiotics affect phagocytosis and synthesis of pro-inflammatory cytokines, and thus have been proposed as modulators of the allergic response and advocated as therapeutic and preventive interventions for allergic diseases (35, 36). Prebiotics are “non-digestible food components that selectively promote the growth of intestinal microbes with positive effects on host health” (37, 38), specifically stimulating the expansion of bifidobacteria and lactobacilli species (39). Altering the intake with foods of these components or supplementing the diet with prebiotics can modify in a positive manner the proportions and the activity of certain intestinal microbial species (39). Synbiotics are “combinations of prebiotics and probiotics” (40).

The aim of this narrative review was to provide researchers an updated overview on the use of prebiotics, probiotics, and synbiotics for the primary prevention of allergy in infants, highlighting the controversies, current research gaps, and potential developments in the field. This review considered the administration of probiotics as supplements, excluding the possible exposure through common food, naturally containing probiotics (such as fermented milk, yogurt). We searched the Cochrane library and PubMed, (Embase, Medline,) during the last 20 years, up to March 2020, using as keywords the following: “allergic diseases,” “food allergy,” “allergy prevention,” “allergic proctocolitis,” “atopic dermatitis,” “wheezing,” “eczema,” “allergic rhinitis,” “atopic disease,” “prebiotics,” “probiotics,” “synbiotics,” “prevention.” We limited our evaluation by age (“children,” “aged 0–2 years”) and languages (English); however, to be more inclusive, the operators “AND” “OR” were also used. Intervention controlled trials, reviews, meta-analyses, and guidelines on prebiotics and probiotics were considered, as well as the following populations for possible supplementation with probiotics: pregnant women, breastfeeding mothers, and infants, regardless of exclusive breastfeeding. All types of prebiotics and probiotics and doses of supplementation were evaluated.

## Mechanisms of Probiotics/Prebiotics in Modulating Gut Microbiota

Diet is recognized as one of the most important factors which may modulate the gut microbiota composition and function (41). It is well-known that changes in the composition of the diet, such as westernized high fat and low fiber diet, can modify the prevalence and types of intestinal microbial species, as certain species are more suitable to utilize specific substrates (42). An association between low-fiber diet and non-communicable chronic diseases, including allergic diseases have been hypothesized on the basis of observational studies (43). The positive effects of the diet on gut microbiota are hypothesized to be due to the prebiotic component.

As anticipated, prebiotics “are non-digestible food components” that selectively trigger the growth in the gut of microbes with positive effects for host health (38): not being the target of upper gastrointestinal digestive enzymes, they typically reach intact the colon, where they are fermented by intestinal microbes (main endproducts of their fermentation are short-chain fatty acids) and selectively stimulate the growth of those intestinal microorganisms (bifidobacteria and lactobacilli species) that are associated with host health and well-being (44). Indeed, they are the favorite meal of the saccharolytic bacteria living in the human gut, as different bacteria prefer other energy sources. Prebiotics are naturally contained in cereals, fruits, vegetables, etc. (non-digestible oligosaccharides), or can be produced by industry (38, 45). Consequently, by modifying the intake of foods containing these products or by supplementation with prebiotics, diet can be used as a powerful tool to direct the gut microbial population (46, 47).

Firstly, prebiotics are naturally present in human milk, who contains at least 200 human milk oligosaccharides (HMO), while oligosaccharides are virtually absent from cow's milk, which explains the increase of gut bifidobacteria observed in breastfed infants compared with standard formula-fed (SF) (48, 49). Human milk oligosaccharides (among the widest components in human milk together with lactose and fats), may represent an excellent meal for beneficial species and prevent the adhesion of pathogens, contributing to the shift of the infant gut microbiota, influencing the immune system (50) and infants health (51). Different HMOs have different properties and functions (52); their molecular structure differs in size and sequence among women (53), being influenced by certain factors (lactation period, secretor status, maternal Lewis Blood Group, etc.), and giving the infant a different degree of protection. A recent study showed infants receiving human milk with a low Lacto-N-fucopentaose III (LNFP) content were more prone to develop Cow's Milk allergy (CMA) compared to infants fed with milk containing a high concentration of LNFP III (OR: 6.7, 95% CI 2.0–22) (54). However, the role of breastfeeding (BF) in preventing allergic diseases is still debated (55), with studies showing no protective effect or even an increased risk for AD with prolonged exclusive breastfeeding (40, 56–58), while other studies/systematic reviews reporting positive effects on prevention, mostly of AD (39, 59). From a mechanistic point of view, BF is thought to prevent allergy development through its content of allergens and immune mediators, absent in artificial milk (55, 60), as well as of HMOs known to stimulate a gut microbiota that might induce tolerance (61). Consequently, when breastfeeding is not possible, trying to reproduce the functional effects of HMOs, infant formulas have been supplemented with galactooligosaccharides and/or fructooligosaccharides. Studies in term and preterm infants indicate that a short-chain galacto-oligosaccharides (scGOS)/long-chain fructo-oligosaccharides (lcFOS) mixture has prebiotic activities, producing a gut microbiota similar to that of breastfed infants (62–64).

Moreover, in murine models, galacto-oligosaccharides (GOS) have been shown to improve the skin lesions of atopic dermatitis, by inducing the production of IL-10 and blocking the production of pro-inflammatory cytokines, such as IL-17, (65). In addition, prebiotics were reported to decrease Ig free light chain (Ig-fLC) concentrations in infants at high-risk for allergies (66): Ig-fLC might play a role in the pathophysiology of the allergic disease since increased Ig-fLC content was found in patients with AD, allergic rhinitis, asthma or cow's milk allergy. However, overall, the mechanism of action of prebiotics seems mostly due to the previously described indirect effects on gut microbiota.

Another approach used for shaping gut microbiota is the supplementation of the diet with probiotics. Among ligands for “pattern recognition receptors,” Toll-like receptors (TLRs), able to activate the immune system, such as virus and the recently identified virus-derived synthetic RNA-DNA hybrids, Bacteria are considered the most powerful immunomodulating factors (39).

The possible mechanism of action of probiotics in this regard includes influences on the maturation of intestinal barrier and on immune response by rebalancing Th1 and Th2 response while suppressing Th17 cells, promoting Tolerogenic Dendritic and Regulatory T (Treg) cell development, and pattern recognition receptor (TLR) stimulation (67). In fact, dendritic cells within the gut mucosa play a key role in the differentiation of regulatory T cells (Treg) which are known to be important in the development of immune tolerance (68). Alterations in Treg functions are associated with the development of allergic diseases (69) and evidence indicates that the gut microbiota acquired early in life is essential for the right development of Treg and Th1/th2 balance (70). The possible mechanisms whereby probiotics may obtain atopy prevention include the stimulation of Th1 response and a decrease in the secretion of Th2 cytokines, such as interleukin (IL)-4, IL-5, and IL-13, a decrease in IgE, and a rise of C-reactive protein and IgA (41). In addition, a murine model of asthma showed that neonatal supplementation with probiotics inhibits the development of allergic sensitization and airway disease by inducing regulatory T cells (Tregs) and producing transforming growth factor-B (71).

Indeed, selected strain of probiotics (such as *Lactobacillus* GG) provides maturational signals for the gut-associated lymphoid tissues (GALT) and development of Tolerogenic dendritic and regulatory T (Treg) cell differentiation, which will induce intestinal barrier maturation and reduce the prevalence of the allergic reactions (72). Therefore, by improving the barrier function and reducing the leakage of antigens through the mucosa probiotics may reduce the potential exposure to allergens (73). Moreover, specific probiotics demonstrated local and systemic anti-inflammatory effects referred to increased secretion of IL-10 (67). Other researchers suggested as a possible mechanism of action of probiotics, in regard to protection against allergic diseases, also the stimulation of Toll-like receptors, which induce the production of mediators, e.g., IL-6, and further IgA secretion (74).

In addition, through increased production of secretory IgA, which contributes to the exclusion of antigens from the intestinal mucosa (75) probiotics may obtain direct modulation of the immune system and eventually prevention of allergic diseases (76).

Moreover, colonizing the mother pre-natally by probiotics supplementation, together with subsequent changes in her breast milk composition and cytokines pattern, with an increased concentration of transforming growth factor-beta (TGF-b), could be beneficial for the infant regarding allergy development (77) and acquisition of immunotolerance (78).

In summary, the probiotics could potentially produce local effects, such as permeability reduction and thus systemic antigens penetration, increased local IgA production, and tolerance induction. Moreover, anti-inflammatory effects mediated by Toll-like receptors, the stimulation of Th1 response to allergens, the activation of tolerogenic dendritic cells, and the production of Treg are among the systemic effects of probiotics (75, 79).

However, despite the evidence on possible mechanisms of action of different preventive strategies, studies evaluating the efficacy of prebiotic and/or probiotic supplementation in the prevention of allergic diseases have yielded conflicting results.

## Prebiotics to Prevent Allergic Diseases

The bifidogenic effect of human milk (rich in oligosaccharides) is well-known. Prebiotics have long been added to infant milk formulas to mimic these functional characteristics of breast milk (52, 80, 81). A combination of galacto-oligosaccharide (GOS) and fructo-oligosaccharide (FOS) (scGOS 90% plus lcFOS 10%) was prebiotic of choice in a number of intervention trials. Acidic oligosaccharides (AOS), polydextrose (PDX) (with or without lactulose), different content of lactose, oligofructose plus inulin have also been tested (Table 1). Modification of intestinal microbiota represents the principal way by which this effect has been orchestrated (93) and has been reported in several studies (82, 90, 92, 94, 95). The 2′-fucosyllactose (2′-FL) human milk oligosaccharide (HMO), the most plentiful HMO in most human milk, has been recently synthesized and is now commercially available in few supplemented infant formulas, bringing the composition closer to human milk (95).

**Table 1:** Prebiotics administration in prevention of allergic disorders.

**(A)** Prebiotic + Standard formula (or prebiotic of human milk).						
**References**	**Study**	**Enrolled patients**	**Prebiotic** **+** **Standard formula (or prebiotic of human milk)**	**Prebiotic substance**, **Beginning of treatment (S)**, **End of treatment (E)**.	**Outcomes**	**Follow-up (duration)**
Ziegler et al. (82)	double-blind, randomized, controlled, parallel-group, prospective trial	226 healthy term infants in 3 groups: - 58 in control group: control formula only - 58 in PG4 group: control formula +4 g/L prebiotic mixture - 48 in PGL8 group: control formula +8 g/L prebiotic mixture	Control formula added with a prebiotic mixture (4 g/L) of PDX and GOS, 50:50 ratio (PG4 group) Control formula containing a prebiotic mixture (8 g/L) of PDX, GOS, and LOS, 50:33:17 ratio). (PGL8 group)	S: 14 days of age E: 120 days of age	Infants fed formula containing a prebiotic mixture achieved normal growth and stool characteristics more similar to those of breast-fed infants (softer, looser) compared to infants fed an unsupplemented formula. Statistical difference among adverse events: - Eczema (PG4 vs. control: 18 vs. 7%, *P* = 0.046; PG4 vs. PGL8: 18 vs. 4%, *P* = 0.008) - Diarrhea: control vs. PG4: 4 vs. 18%, *P* = 0.008) - Irritability: control vs. PGL8, 4 vs. 16%, *P* = 0.027)	120 days
Niele et al. (83)	Double-blind, randomized placebo controlled trial	113 preterm infants (GA < 32 weeks or Wt < 1.500 gr) 94/98 infants eligible at the corrected age of 1 year participated in the follow-up study	Prebiotic mixture: 80% scGOS/lcFOS and 20% pAOS Placebo mixture: maltodextrin in increasing dose for 30 days. After discharge, all infants received Human Milk or Nenatal Start or Nenatal 1 (both without oligosaccharides or probiotics)	S: < 3days of life E: 30 days of life	Supplementation with non-human neutral and acidic oligosaccharides during the neonatal period in preterm infants did not significantly decrease the incidence of allergic and infectious diseases during the 1st year of life (AD at 1 year: 15 vs. 19%)	12 months
Gruber et al. (84)	double-blind, placebo-controlled, randomized prospective nutritional intervention study	Healthy term infants with low atopy risk: - 414 infants in prebiotic group (PG) - 416 infants in control group (CG). - 300 infants in breast-feeding group (BG)	PG: regular formula containing a specific mixture of neutral oligosaccharides [scGOS/lcFOS, ratio 9:1, (85 wt%),] and pectin-derived acidic oligosaccharides OS) (15wt%) CG: Standard formula without oligosaccharides. BG: Breast milk	S: before post-natal age of 8 weeks E: 12 months	Formula containing a mixture of neutral oligosaccharides was effective as primary prevention of atopic dermatitis in low atopy risk infants (5.7% in PG vs. 9.7% in CG, *P* = 0.04; 7.3% in BG)	1 year
Gruber et al. (85)	double-blind, controlled, randomized prospective nutritional intervention study	Healthy term infants with low atopy risk: - 232 infants in prebiotic formula group (PG) - 243 infants in control formula group (CG) - 197 infants in breast-feeding group (BG)	PG: regular formula containing aspecific mixture of neutral oligosaccharides [scGOS/lcFOS, ratio 9:1, (85 wt%),] and pectin-derived acidic oligosaccharides OS)(15wt%) CG: Standard formula without oligosaccharides. BG: Breast milk	S: before post-natal age of 8 weeks E: 12 months	The cumulative incidence of AD up to age 5 years was 18.2% (PG) 20.2% (CG) and 23.9% (BG), therefore in this follow up study there was no sustained statistically significant effect of prebiotics added to infant diet against the occurrence of early AD at preschool age	5 years
Pontes et al. (86)	double-blind, randomized, controlled trial	healthy children (1–4 years of age) 125: CMBB with DHA,PDX,GOS, β-glucan, and other key nutrients 131: control	Cow's Milk-Based Beverage (CMBB) containing DHA, the prebiotics polydextrose (PDX) and galactooligosaccharides (GOS), β-glucan, and other nutrients including zinc, vitamin A and iron	S: 1–4 years of age E: 28 weeks later	CMBB was associated with fewer episodes of allergic manifestations (atopic dermatitis, wheezing, allergic rhinitis) compared to controls (*p* = 0.021)	28 weeks.
Ranucci et al. (87)	randomized, double-blind, placebo-controlled trial	118/201 infants who received a prebiotic (GOS/PDX)-enriched formula (PF) completed the study 104/199 infants who received an SF until 48 weeks of life completed the study 123/140 infants who remained on exclusive breastfeeding until six months of age completed the study	prebiotic (mixture of 4 g/L of GOS/PDX)-enriched standard formula (PF) vs. identical standard formula without prebiotic	S: birth E: 48 weeks of life	There were not significant differences in the cumulative incidence, intensity and duration of AD among groups. However, the risk of AD in PF was reduced by 35% compared with SF. Bifidobacteria and Clostridium clusterI colonization increased in the PF group. Bifidobacteria was associated with RIs protection, whereas Clostridium cluster I had a protective role in atopy development	96 weeks
**(B)** Prebiotic +Hydrolyzed/ amino acid-based formulas.						
**References**	**Study**	**Enrolled patients**	**Hydrolyzed/ amino acid-based formulas+** **Prebiotic substance**	**Prebiotic substance**, **Beginning of treatment (S)**, **End of treatment (E)**.	**Outcomes**	**Follow-up (duration)**
Moro et al. (80)	Prospective randomized, double-blind placebo controlled trial	206/259 infants at high risk of atopy completed the study: 102 infants in the prebiotic group; 104 infants in the placebo group	Extensively hydrolysed cows'milk whey protein formula supplemented either with 8 g/L scGOS/lcFOS / (prebiotic group) or a 8 g/L maltodextrin (placebo group)	8 g/L scGOS/lcFOS S: within the first 2 weeks of life E: 6 months	The cumulative incidence of AD atopic dermatitis was significantly reduced at 6 months of age by prebiotics supplementation (9.8 vs. 23.1%, *p* < 0.05)	6 months
Arslanoglu et al. (88)	Prospective randomized, double-blind placebo controlled trial	134/152 infants at high risk of atopy completed the study 66 in the prebiotic group 68 in the placebo group	Extensively hydrolysed cows'milk whey protein formula supplemented either with 8 g/L scGOS/lcFOS / (prebiotic group) or a 8 g/L maltodextrin (placebo group).	8 g/L scGOS/lcFOS S: within the first 2 weeks of life E: 6 months	Cumulative incidences of AD, recurrent wheezing, and allergic urticaria were significantly reduced at 2 years of age by prebiotics supplementation (13.6%, 7.6%, and 1.5 vs. 27.9%, 20.6% and 10.3% respectively, *p* < 0.05)	2 years
Arslanoglu et al. (89)	Prospective randomized, double-blind placebo controlled trial,	92 infants at high risk of atopy completed the study 42 in the prebiotic group 50 in the placebo group	Extensively hydrolysed cows'milk whey protein formula supplemented either with 8 g/L scGOS/lcFOS / (prebiotic group) or a 8 g/L maltodextrin (placebo group).	8 g/L scGOS/lcFOS S: within the first 2 weeks of life E: 6 months	Cumulative incidences of any allergic manifestations and atopic dermatitis were significantly reduced at 5 years of age by prebiotics supplementation (30.9%, and 19.1 vs. 66 and 38%, respectively, *p* < 0.05)	5 years
Francavilla et al. (90)	Prospective two-phases clinical trial (cross-over design)	21 infants with a confirmed CMA 15 healthy breast-fed infants as controls	Phase 1: extensively hydrolyzed formula without lactose for 2 months Phase 2: an identical extensively hydrolyzed formula containing lactose (3.8%) for an additional 2 months	3.8% Lactose	The addition of lactose to an extensively hydrolyzed formula increased the total fecal counts of Lactobacillus/Bifidobacteria, the concentration of total short-chain fatty acids, mostly acetic and butyric acids and decreased the counts of Bacteroides/Clostridia	4 months
Boyle et al. (91)	double-blind, randomized, controlled parallel-group nutritional intervention trial	863 high-risk infants: - 432 infants in the prebiotic group (PG) - 431 Infants fed with standard foumula (CG	PG: partially hydrolysed whey-based infant formula containing a specific mixture of neutral scGOS and lcFOS (9: 1; 85 weight per cent, 0.68 g/100 ml) and acidic pAOS (15 weight per cent, 0.12 g/100 ml acidic) (pHF-OS)	S: before 18 weeks of life E: 6 months	pHF-OS did not prevent eczema in high-risk infants in the first 12 months (Eczema occurred in 30.8% pHF-OS vs. 30.3% control in all infants (OR 0.99 95% CI 0.71, 1.37; *P* = 0.94). as well as by 18 months. However, pHF-OS reduced cow's milk-specific IgG1 (*P* < 0.0001)	12 and 18 months
Wopereis et al. (92)	Double-blind, randomized, controlled, parallel group nutritional intervention trial	138 Infants at high risk: - 51 infants in the prebiotic group (PG) - 57 Infants fed with standard foumula (CG) - 30 infants in the breast-fed group (BG)	PG: Partially hydrolyzed formula containing short-chain galacto-oligosaccharides and long-chain fructo-oligosaccharides (9:1; 0.68 g/100 mL) and pectin-derived acidic oligosaccharides (0.12 g/100 mL) CG: standard formula BG: breast milk	S: before 18 weeks of life E: 26 weeks of age	Infants with eczema at 18 months: 32% in CG, 39% in PG and 47% in BG Infants presenting eczema at 18 months showed a decrease in acquisition of *Eubacterium* and *Anaerostipes* species with increased lactate and reduced butyrate levels	18 months

*Cow's milk protein allergy (CMA), Healthy controls (HC), Human milk oligosaccharides (HMO), short-chain galactooligosaccharides (scGOS), long-chain fructooligosaccharides(lcFOS), human milk oligosaccharides (HMOS), prebiotics polydextrose (PDX), galactooligosaccharides (GOS), galacto-oligosaccharide/polydextrose (GOS/PDX), Probiotic formula (PF), atopic dermatitis (AD), cow's milk-based beverage (CMBB)*.

*Standard formula (SF), Breast feeding (BF)*.

We summarize the evidence on the preventive effects of different prebiotic administration in Table 1. The majority of these studies evaluated the effects of prebiotics on atopic dermatitis (AD); other allergic manifestations were much less investigated; however, it remains unclear whether prebiotics supplementation can prevent allergic diseases due to heterogeneity of the studies and type of prebiotics. In a longitudinal cohort study enrolling 259 high-risk infants, Moro et al. (80) found that a hydrolyzed protein cow's milk-based formula supplemented with 90% scGOS−10% lcFOS, (8g/L) significantly reduced AD at the age of 6 months [intervention group: 9.8 vs. 23.1% placebo group (*P* < 0.05)] and increased the number of fecal bifidobacteria. Long term beneficial effect on allergy prevention (i.e., atopic dermatitis, rhinoconjunctivitis, and allergic urticaria) was also noted during the follow-up period, at 2 and 5 years of age compared to the placebo group (88, 89). In another RCT study (84) a 44% lower incidence of AD was reported at 1 year of life in infants at low risk of allergy fed an intact protein formula supplemented with GOS/FOS and specific pectin-derived acidic oligosaccharide compared to infants fed standard formula. It is noteworthy that the rate of AD in the prebiotic group was similar to that of fully breastfed babies (5.7 vs. 7.3%) but the protective effect vanished at preschool age (85).

Supplementation with prebiotics also showed a beneficial effect when used in children aged 1–4 years old. In a double-blind, randomized, controlled trial (86), 125 children were given cow's milk-based beverage (CMBB) containing DHA, the prebiotics polydextrose (PDX) and galactooligosaccharides (GOS), beta-glucan, zinc, iron, vitamins A and D, and were compared to 131 children fed with standard cow's milk for 28 weeks. Children who consumed CMBB had significantly reduced episodes of allergic manifestation, including eczema and urticaria, allergic rhinitis or conjunctivitis, wheezing, and allergic cough, compared to the control group. A meta-analysis (96) evaluating different types of prebiotics, duration of administration, and length of follow-up concluded for an overall 32% reduced risk of eczema and dermatitis (RR:0.68, 95% CI: 0.48–0.97; NNT 25), but not of asthma.

However, other trials did not confirm these positive results (83, 87, 91, 97–100). In a study (83), evaluating preterm, low birth weight infants fed with a formula containing a prebiotic mixture (GOS/FOS plus acidic oligosaccharides), there was no difference in the prevalence of AD and bronchial hyper-reactivity. In another study (91) a partially hydrolyzed formula supplemented with specific oligosaccharides (pHF-OS) induced immunomodulatory effects, such as increased regulatory T-cell numbers, in infants at increased risk of allergy, but was not able to reduce AD incidence at 12 or 18 months compared with standard formula-fed infants.

In 2011, the Nutrition Committee of the European Society for Pediatric Gastroenterology Hepatology and Nutrition (ESPGHAN) found insufficient evidence to recommend supplementing with prebiotics in infant formulas to prevent atopic disease (98).

In 2016, the World Allergy Organization (99), using the Grading of Recommendations Assessment, Development, and Evaluation (GRADE) approach, was in favor to use prebiotic supplementation in not-exclusively breastfed infants but reporting very low certainty of evidence. No significant difference in eczema (RR: 0.57, 95% CI: 0.30–1.08) emerged after the meta-analysis of five studies (1,313 infants), while the meta-analysis of two studies (249 infants) found a reduction in recurrent wheeze or asthma (RR: 0.37, 95% CI: 0.17–0.80) in the prebiotic group of infants. Only one study assessed the risk of food allergy and found a reduced risk (RR: 0.28, 95% CI 0.08–1.00) in infants supplemented with prebiotics.

In 2017 a systematic review performed by Cuello-Garcia et al. (100) found not enough evidence to reject or to support the use of prebiotics for allergy prevention in infants analyzing the risk of AD (RR: 0.68, 95% CI: 0.40–1.15), asthma/wheezing (RR, 0.37; 95% CI: 0.17–0.80), and food allergy (RR: 0.28, 95% CI: 0.08–1.00). No evidence of an increased risk of any adverse effects was also noted in supplemented infants (RR: 1.01, 95% CI: 0.92–1.10). In 2018 infants with a positive family history of allergy were randomized to receive a GOS/PDX-formula (PF) or standard formula (SF) until 48 weeks of life while 140 infants were exclusively breastfed (BF): even if there was a 35% reduction in AD risk in PF compared with SF, there was no a statistically significant difference in any AD analyzed variables between the two groups at 36, 48, and 96 weeks (87).

Interestingly, in the same year, a systematic review (101) on HMOs reported a preventive effect on cow's milk allergy (CMA) at 18 months of age.

Therefore, at present, despite some promising results with specific prebiotics on the gut microbiota (102), the heterogeneity and the limited numbers of studies do not allow to draw any definitive conclusions on the clinical impact of prebiotics for allergy prevention.

As it has been suggested (103), since a large amount of prebiotics are already present in human milk, more carefully conducted RCTs in formula-fed infants, at high as well at low risk of allergy, are still needed before routine prebiotic supplementation can be recommended for allergy prevention.

## Probiotics to Prevent Allergic Diseases

Recent evidence suggests that exposure to beneficial bacteria in early life may have a role in the prevention of allergy (72). A number of studies first demonstrated that infants born vaginally and breastfed are colonized by Lactobacilli and Bifidobacteria whilst infants born through cesarean section and fed with standard formula show a significantly lower prevalence of Bifidobacteria and more Bacteroides and Coliforms (72) associated with increased prevalence of respiratory allergies (104). Thus, probiotic supplementation during pregnancy was considered to transfer beneficial bacteria to the infant during delivery and after birth. Secondly, the gut is highly exposed to microbial exposure and immune stimulation (105) and probiotic supplementation early in life may facilitate the maturation of the immune system (106, 107). Based on these hypotheses, most trials evaluating probiotics for prevention of allergy are based on supplementation during pregnancy, lactation; and/or post-natally. The route of administration varied from oral preparation (capsules; oil droplets; and suspension), addition to infant formula, maternal intake in breastfed babies, or a combination of the above (108). Various microbial species have been tested, in primis *Lactobacillus* and *Bifidobacterium*, alone or in combination with other species, such as *Propionibacterium, Streptococcus, Lactococcus*, and *Escherichia coli* (Table 2).

**Table 2:** Probiotics administration in prevention of allergic disorders.

**(A)** Probiotic given orally (eg droplets, suspensions, capsules) or with breastfeeding/ standard formula.								
**References**	**Study**	**Enrolled patients**	**Probiotic** **+** **Standard formula/breast Feeding**	**Probiotic strain**, **Beginning of treatment (S)**, **End of treatment (E)**.	**Pre-natal administra-tion (duration)**	**Post-natal administration (duration)**	**Outcomes**	**Follow-up (duration)**
Kalliomaki et al. (109)	double-blind, randomized, placebo-controlled trial	− 159 Pregnant woman who had at least one first-degree relative (or partner) with atopic disease - breastfeeding mothers - their infants, post-natally if not breast-fed	Placebo group (*n* = 82): two capsules of placebo (microcrystalline cellulose) Probiotic group (*n* = 77): two capsules of 1 × 10^10^ CFU of *Lactobacillus* GG daily: for infants contents were mixed with water and given by spoon	Pregnant woman: S: 2–4 weeks before expected delivery E: at delivery or 6 months later if breastfeeding mothers Infants: S: birth E: 6 months	2–4 weeks before expected delivery	6 months	There was a halving in frequency of atopic eczema in the probiotic group compared with the placebo group (15/64 [23%] vs. 31/68 [46%]; relative risk 0.51 [95% CI 0.32–0.84]). The number needed to treat was 4.5 (95% CI 2.6–15.6)	2 years
Rautava et al. (111)	parallel, double-blind placebo-controlled trial	205 pregnant women with allergic disease and atopic sensitization	Probiotic groups: - 1 sachet of L.rhamnosus LPR (1 × 109 CFU) and B. longum BL999 (1 × 109 CFU (*N* = 73) daily or - L paracasei ST11 (1 × 109 CFU) and B longum BL999 (1 × 109 CFU) daily (*N* = 70) Placebo group (*n* = 62): the same sachet without probiotics	S: 2 months before expeted delivery E: 2 months after delivery (during breast-feeding)	2 months before expeted delivery to delivery	2 months	There was a significantly reduced risk of developing eczema in infants of mothers receiving LPR1BL999 (odds ratio [OR], 0.17; 95% CI, 0.08-0.35; *P* <.001) and ST111BL999 (OR, 0.16; 95% CI, 0.08–0.35; *P* <.001)	2 years
Kalliomäki et al. (112)	double-blind, randomi-zed, placebo-controlled trial	- 132 Pregnant woman who had at least one first-degree relative (or partner) with atopic disease -breastfeeding mothers - their infants, post-natally if not breast-fed	Placebo group (*n* = 53): two capsules of placebo (microcrystalline cellulose) Probiotic group (*n* = 54): two capsules of 1 × 10^10^ CFU of *Lactobacillus* GG daily: for infants contents were mixed with water and given by spoon	Pregnant woman: S: 2–4 weeks before expected delivery E: at delivery or 6 months later if breastfeeding mothers Infants: S: birth E: 6 months	2–4 weeks before expected delivery	6 months	There was an extention beyond infancy of the preventive effect of lactobacillus GG on atopic eczema: (14/53 in probiotic group developped eczema vs. 25/54 receiving placebo (relative risk 0.57, 95% CI 0.33–0.97)	4 years
Kalliomäki et al. (113)	double-blind, randomized, placebo-controlled trial	- 116 Pregnant woman who had at least one first-degree relative (or partner) with atopic disease -breastfeeding mothers -their infants, post-natally if not breast-fed	Placebo group (*n* = 62): two capsules of placebo (microcrystalline cellulose) Probiotic group (*n* = 53): two capsules of 1 × 10^10^ CFU of *Lactobacillus* GG daily: for infants contents were mixed with water and given by spoon	Pregnant woman: S: 2–4 weeks before expected delivery E: at delivery or 6 months later if breastfeeding mothers Infants: S: birth E: 6 months	2–4 weeks before expected delivery	6 months	The cumulative risk for developing eczema was significantly lower in the L.GG group than in the placebo group (42.6% vs. 66.1%; RR, 0.64; 95% CI, 0.45-0.92) According to Cox regression, the risk of eczema was significantly reduced in the L. GG group (odds ratio, 0.58; 95% CI, 0.35–0.94; *P* = 0.027)	7 years
Wickens et al. (114)	Double-blind, randomized placebo-controlled trial	- Pregnant women who had at least one first-degree relative (or partner) with atopic disease,- breast feeding mothers -their infants	Two Probiotic groups(capsule powder with): - Lactobacillus rhamnosus HN001 (*N* = 170) - Bifidobacterium animalis subsp lactis strain HN019 (*N* = 171) Placebo group: (*N* = 171): capsule powder without probiotics	Pregnant women: Lactobacillus rhamnosus HN001 (6 x 3 109 CFU /d), Bifidobacterium animalis subsp lactis strain HN019 (9 x 3 109 CFU /d) or placebo daily from 35 weeks gestation until 6 months if breast-feeding Infants: same treatment from day 2-16 of life to 2 years	From 35 weeks gestation	Breast feeding mothers: for 6 months Infants: for 2 years since day 2-16 of life	infants receiving L rhamnosus had a significantly (P = 0.01) reduced risk of eczema (hazard ratio [HR], 0.51; 95% CI, 0.30–0.85) compared with placebo, but this was not the case for B animalis subsp lactis (HR, 0.90; 95% CI, 0.58–1.41)	2 years
Dotterud et al. (115)	randomized, double-blind trial	415 pregnant women	Probiotic group (*n* = 138): probiotic milk contained LGG 5 × 1010 CFU, Bb-12 5 × 1010 CFU and La-5. 5 × 109 CFU daily. Placebo group (*N* = 140): the placebo milk contained no probiotic bacteria	S: 4 weeks before expected delivery date E: 3 months after delivery (while breastfeeding)	4 weeks (from 36 weeks of gestation)	3 months while brestfeeding	There was a odds ratio (OR) of 0.51 for the cumulative incidence of AD in the probiotic group compared with the placebo [95% CI, 0.30–0.87; *P* = 0.013]. There were no significant effects on asthma or atopic sensitiza- tion	2 years
Kim et al. (116)	randomized, double-blind, placebo-controlled trial	112 pregnant women and newborns	Probiotics group: mixture of B. bifidum BGN4 [1.6 × 10^9^CFU], B. lactis AD011 (1.6 × 10^9^ CFU), and L. acidophilus AD031 (1.6 × 10^9^ CFU) in 0.72 g of maltodextrin and 0.8 g of alpha-corn once daily Placebo group: maltodextrin and alpha-corn without probiotic bacteria	S (women): 4–8 weeks before expected delivery E (women): 3 months after delivery (during breastfeeding) S (infants): 4 months after delivery E(infants): 6 months	4–8 weeks before expected delivery to delivery	6 months	There was a significant reduction in the cumulative incidence of eczema during the first year in probiotic group (36.4% vs. 62.9%, *p* = 0.029)	1 year
West et al. (117)	double-blind, placebo-controlled randomized intervention trial	179 infants during weaning	Probiotic group (*n* = 89): fed cereals with *Lactobacillus* F19 Placebo group(*N* = 90): fed cereals without probiotics	S: 4 months E: 13 months	no	9 months	There was a cumulative incidence of eczema of 11% (4–17%, 95% CI) in the probiotic group vs. 22% (13–31%, 95% CI) in the placebo group (*p* < 0.05)	13 months
Lodinova-Zadnikova et al. (118)	controlled clinical trial	158 infants: - *N* = 56 colonized infants of allergic mothers, *N* = 57 control infants of allergic mothers - *N* = 45 control infants of healthy mothers	One milliliter of *E. coli* was administered to infants of allergic mothers	S: within 48 h after birth and subsequently 3 times a week E: 4 weeks	no	4 weeks	There were allergy symptoms in 14 infants of control allergic mothers, in 7 infants of healthy mothers, and in 2 colonized infants of allergic mothers	5 years
Ezaki et al. (119)	Retrospective study	30 newborns after small intestine surgery	Probiotic group (*N* = 18 newborns GA 34.5 (23.5–38.4): suspension of *B. breve* (7.5 × 10^8^ cells/day). Placebo group (*N* = 12 newborn, GA 34.4 (26.4–40.0):	S: After small intestine sugery E: when full enteral feeding (100 ml/kg/day) was reached	no	After small intestine surgery until full enteral feeding (100 ml/kg/day) was reached	Administration of probiotics reduced the incidence of cow's milk protein intolerance (CMPI) after small intestine surgery (one vs. eight, *p* < 0.001)	
Wickens et al. (120)	Double-blind, randomized placebo-controlled trial	- Pregnant women who had at least one first-degree relative (or partner) with atopic disease, -breast feeding mothers -their infants (*N* = 425)	Two Probiotic groups: - Lactobacillus rhamnosus HN001 - Bifidobacterium animalis subsp lactis strain HN019 Placebo group:	Pregnant women: Lactobacillus rhamnosus HN001 (6 × 3 109 CFU/d), Bifidobacterium animalis subsp lactis strain HN019 (9 × 3 109 CFU/d) or placebo daily from 35 weeks gestation until 6 months if breast-feeding Infants: same treatment from day 2-16 of life to 2 years	From 35 weeks gestation	Breast feeding mothers: for 6 months Infants: for 2 years since day 2–16 of life	The cumulative prevalence of eczema [Hazard ratio (HR) 0.57 (95% CI 0.39–0.83)] and prevalence of rhinoconjunctivitis [Relative risk 0.38 (95% CI 0.18–0.83)] were significantly reduced in the children taking HN 001; HN 019 did not affect the prevalence of any outcome	4 years
Wickens et al. (121)	Double-blind, randomized placebo-controlled trial	- Pregnant women who had at least one first-degree relative (or partner) with atopic disease, -breast feeding mothers -their infants (*N* = 425)	Two Probiotic groups: - Lactobacillus rhamnosus HN001 - Bifidobacterium animalis subsp lactis strain HN019 Placebo group:	Pregnant women: Lactobacillus rhamnosus HN001 (6 × 3 109 CFU/d), Bifidobacterium animalis subsp lactis strain HN019 (9 × 3 109 CFU/d) or placebo daily from 35 weeks gestation until 6 months if breast-feeding Infants: same treatment from day 2-16 of life to 2 years	From 35 weeks gestation	Breast feeding mothers: for 6 months Infants: for 2 years since day 2–16 of life	HN001 was associated with significantly lower cumulative prevalence of eczema (HR = 0.56, 95% CI 0.39–0.80), SCORAD ≥ 10 (HR = 0.69, 0.49-0.98) and SPT sensitization (HR = 0.69, 95% CI 0.48–0.99). HN019 had no significant effect on any outcome	6 years
Wickens et al. (122)	Double-blind, randomized placebo-controlled trial	- Pregnant women who had at least one first-degree relative (or partner) with atopic disease, -breast feeding mothers -their infants	Two Probiotic groups: - Lactobacillus rhamnosus HN001 (*N* = 97) - Bifidobacterium animalis subsp lactis strain HN019 (*N* = 104) Placebo group: (*N* = 97) The capsule powder was either given undiluted to the infant or mixed with water, breast milk, or formula and given via a teaspoon or syringe or sprinkled on food.	Pregnant women: Lactobacillus rhamnosus HN001 (6 × 3 109 colony-forming units/d), Bifidobacterium animalis subsp lactis strain HN019 (9 × 3 109 colony-forming units/d) or placebo daily from 35 weeks gestation until 6 months if breast-feeding Infants: same treatment from day 2-16 of life to 2 years	From 35 weeks gestation	Breast feeding mothers: for 6 months Infants: for 2 years since day 2–16 of life	HN001 significantly reduced the 12-month prevalence of eczema at age 11 years (relative risk [RR] = 0.46, 95% CI 0.25-0.86, *P* = 0.015) and hay fever (RR = 0.73, 95% CI 0.53–1.00, *P* = 0.047). HN001 was associated with a significant reduction in lifetime prevalence of atopic sensitization (hazard ratio [HR] = 0.71, 95% CI 0.51–1.00, *P* = 0.048), eczema (HR = 0.58, 95% CI 0.41–0.82, *P* = 0.002) and wheeze (HR = 0.76, 95% CI 0.57–0.99, *P* = 0.046). HN019 had no significant effect	11 years
Bertelsen et al. (123)	large, prospecti-ve pregnancy cohort study	40,614 mothers and children	probiotic milk products containing *L. acido-philus* LA-5, *B. lactis* Bb12, +/- *L. rhamno-sus* GG	S(mother): during pregnancy S(infants): after 6 months E: 18 months	during pregnancy	Mothers: during breast-feeding Infants: from 6 to 18 months of age	Consumption of probiotic milk in pregnancy was associated with a slightly reduced risk [(adjusted RR (aRR)] of atopic eczema at 6 months aRR=0.94 (95% CI: 0.89, 0.99) and of rhinoconjuctivitis between 18 and 36 months, aRR=0.87 (95% CI: 0.78, 0.98); the adjusted relative risk of rhinoconjunctivitis was aRR=0.80 (95% CI: 0.68, 0.93) when both mother and infant had consumed probiotic milk	36 months
Simpson et al. (124)	Double-blinded, randomized placebo-controlled trial	161 pregnant women	Probiotic group (*N* = 81): probiotic milk contained LGG 5 × 1010 CFU, Bb-12 5 × 1010 CFU and La-5. 5 × 109 CFU daily. Placebo group (*N* = 80): the placebo milk contained no probiotic bacteria	S: 4 weeks before expected delivery date E: 3 months after delivery (while breastfeeding)	4 weeks (from 36 weeks of gestation)	3 months while brestfeeding	There was a trend toward a lower cumulative incidence of AD in the probiotic group (OR 0.64, 95 % CI 0.39–1.07, *p* = 0.086; NNT = 10). This finding was statistically significantly in the complete case analysis (OR 0.48, 95 % CI 0.25–0.92, *p* = 0.027, NNT = 6)	6 years
Schmidt et al. (126)	double-blind, placebo-controlled intervention trial	290 infants aged 8 to 14 months (Mean age 10.1 months)	Probiotic group (*N* = 144): B. animalis subsp lactis and L. rhamnosus (10^9^ CFU of each) daily + maltodextrin powder Placebo group (*N* = 146): maltodextrin powder	S: up to 12 weeks before expected start in child care. E: after 6 months	no	6 months	A significantly lower incidence of eczema was observed in the probiotic group compared to the placebo group (4.2% vs. 11.5%, *P* = 0.036). The incidence of asthma, rhinitis, conjunctivitis, and sensitization did not differ	6 months
Peldan et al. (127)	double-blinded, placebo-control-led study	1223 mothers with infants at high risk for allergy	445 mothers received probiotic‘s mixture: LGG (5 × 10^9^ cfu), L rhamnosus LC705 (5 × 10^9^ cfu), B. breve Bb99 (2 × 10^8^ cfu), and Propionibacterium freudenreichii ssp. shermanii JS (2 × 10^9^ cfu) twice daily. Their infants received the same probiotic capsule + syrup containing 0.8 g of galacto-oligosaccharides once daily 446 mothers and infants received capsules containing microcrystalline cellulose, (placebo) and the infants also received syrup without galacto-oligosaccharides	S (women): From 36 weeks of gestation, E (women): at delivery S (infants): birth E (infants): 6 months	From 36 weeks of gestation,	from birth until age 6 months.	the prevalence of allergic rhino-conjunctivitis was greater in the probiotic group compared to the placebo group (36.5% vs. 29.0%, OR: 1.43, 95% CI: 1.06–1.94, *p* = 0.03	5-10 years
Taylor et al. (128)	Randomized, double-blind, placebo-controlled trial	178 newborns at high risk of allergy: - Probiotic group (*n* = 89) - Placebo group(*n* = 89)	Probiotic group: 3 × 10^9^ L. acidophilus LAVRI-A1 once a day(in sachet packets) Placebo group: Maltodrexine	S: births E: 6 months	no	6 months	Early probiotic supplementation with L acidophilus did not reduce the risk of AD at 12 months of age (38/88 vs. 34/87 in the placebo) and was associated with increased allergen sensitization (35/88 vs. 21/86)	12 months
Abrahamsson et al. (129)	prospective double-blind, placebo-controlled, multicenter trial	188 mothers with allergic disease Their infants continued with the same product	Probiotic group: oil + L reuteri ATCC 55730 (1 × 10^8^ CFU) daily Placebo group: (CFUs): the same oil without probiotics	S (Women): 36 weeks of gestational age E (women): delivery S (infants): at birth E (infants):12 months	from gestational week 36 until delivery.	12 months	The cumulative incidence of eczema was similar, 36% in the treated vs. 34% in the placebo group. The probiotic group had less IgE-associated eczema during the second year, 8% vs. 20% (*P* = 0.02),	2 years
Kopp et al. (130)	Randomized, Double-Blind, Placebo-Controlled Trial	- 94 pregnant women with a family history of atopic disease - 89 breastfeeding mothers -their infants (*n* = 94: 5 not breastfeed infants from birth, 89 from the age of 3 months)	L-GG group: 1 capsule(5 × 10^9^ CFU) of L- GG twice Daily (*N* = 50) Placebo group: capsules of microcrystalline cellulose (*N* = 44)	S (women): 4 to 6 weeks before expected delivery, then during breastfeeding for 3 months; S (infants): 5 infants from birth, 89 from the age of 3 months E(women): at delivery or after 3 months if breastfeeding E (infants): 6 months of age	4-6 weeks	6 months	Supplementation with L- GG neither reduced the incidence of AD (28% vs. 27.3%, *P* = 0.93) nor altered the severity of AD but was associated with an increased rate of recurrent wheezing bronchitis (26% vs. 9.1% *P =* 0.03)	2 years
Prescott et al. (131)	Randomized, double-blind, placebo-controlled trial	153 newborns at high risk of allergy: - Probiotic group (*N* = 74) - Placebo group (*N* = 76)	Probiotic group: 3 × 10^9^ L. acidophilus LAVRI-A1 once a day(in sachet packets) Placebo group: Maltodrexine	S: births E: 6 months	no	6 months	Supplementation with this probiotic did not reduce the risk of dermatitis (31/74, 42%) compared with placebo group (25/76, 34%). There was no significant reduction in any other allergic disease or allergen sensitization	2.5 years
Kuitunen et al. (133)	double-blinded, placebo-control-led study	1223 mothers with infants at high risk for allergy	445 mothers received probiotic ‘s mixture: LGG (5 x10^9^ cfu), L rhamnosus LC705 (5 × 10^9^ cfu), B. breve Bb99 (2 x10^8^ cfu), and Propionibacterium freudenreichii ssp. shermanii JS (2 × 10^9^ cfu) twice daily. Their infants received the same probiotic capsule + syrup containing 0.8 g of galacto-oligosaccharides once daily 446 mothers and infants received capsules containing microcrystalline cellulose, (placebo) and the infants also received syrup without galacto-oligosaccharides	S (women): From 36 weeks of gestation, E (women): at delivery S (infants): birth E (infants): 6 months	From 36 weeks of gestation,	from birth until age 6 months	No significant difference appeared in frequencies of eczema (39.3% vs. 43.3%), atopic eczema (24.0% vs. 25.1%), allergic rhinitis (20.7% vs. 19.1%), or asthma (13.0% vs. 14.1%) between groups. However, less IgE-associated allergic disease occurred in cesarean- delivered children receiving probiotics (24.3% vs. 40.5%; odds ratio, 0.47; 95% CI, 0.23% to 0.96%; *P* 5.035)	5 years
Niers et al. (134)	Double-blind, randomized, placebo-controlled trial	98 pregnant women with a family history of allergic diseases and their infants	Probiotic group (*N* = 50): sachets containing B. bifidum (1 × 109 CFU), B. lactis (1 × 109 CFU), and L. lactis (1 × 109 CFU) daily Placebo group (*N* = 48): rice starch and maltodextran	S: last 6 weeks of pregnancy E: 12 months after delivery (to infants)	last 6 weeks of pregnancy	12 months	Cumulative incidence of eczema at 1 and 2 years was 23/50 (intervention) vs. 31/48 (placebo) and 27 (intervention) vs. 34 (placebo), respectively	2 years
Boyle et al. (135)	Randomized controlled trial	250 pregnant women carrying infants at high risk of allergic disease	Probiotic group: Lactobacillus rhamnosus GG (LGG) 1.8 × 10^10^ CFU/day Placebo group	S: 36 weeks of gestation E: at delivery	From 36 weeks of gestation until delivery	no	Pre-natal probiotic treatment was not associated with reduced risk of eczema (34% probiotic, 39% placebo; RR 0.88; 95% CI 0.63, 1.22) or IgE-associated eczema (18% probiotic, 19% placebo; RR 0.94; 95% CI 0.53, 1.68)	
Ou et al. (136)	randomized, double-blind, placebo-controlled trial	191 pregnant women with atopic diseases, breastfeeding mothers or non-breastfeeding neonates,	Probiotic group (*N* = 95):*LGG* ATCC 53103, 1 × 1010 CFU daily Control group (*N* = 96)	S (women): from the second trimester of pregnancy; E: 6 months after delivery (breastfeeding mothers or non-breast-feeding infants from birth)	From the 24 weeks of gestational age to delivery	6 months	There was no significant difference between the cumulative risk of sensitization and developing allergic disease at the age of first 36 months by log-rank test (*P* = 0.86 and *P* = 0.74, respectively)	3 years
Damm et al. (137)	Controlled interventional cohort study	527 preterm neonates (<30 weeks of gestation)	Probiotic group (*N* = 249): L. rhamnosus GG (1 × 10^9^) and B. animalis subsp. lactis (BB12) (1 × 10^8^) daily Control group (*N* = 278): not treated with probiotics	S: third day of life E: at discharge from hospital,	no	from the third day of life to discharge from hospital	The prevalence of AD was similar in the two groups (20.9% in the probiotic treated group vs. 17.1% in the not treated group, *p* = 0.33)	2-8 years
Laursen et al. (138)	randomized, double-blind, placebo-controlled study	290 infants aged 8 to 14 months	Probiotic group (*N* = 144 B. animalis subsp lactis and L. rhamnosus (10^9^ CFU of each) daily + maltodextrin powde Placebo group (*N* = 146): maltodextrin powder	S: up to 12 weeks before expected start in child care. E: after 6 months		6 months	Probiotic treatment did not reduce the number of days absent from child care due to infections in healthy infants at the time of enrollment in child care	6 months
Murphy et al. (139)	Sub-Sample Analysis From a randomized, controlled, 3-arm trial (115, 116)	- Pregnant women who had at least one first-degree relative (or partner) with atopic disease, -breast feeding mothers -their infants	Two Probiotic groups: - Lactobacillus rhamnosus HN001 (*N* = 285 stools) - Bifidobacterium animalis subsp lactis strain HN019 (*N* = 50 stools) Placebo group: (*N* = 315 stools sample)	Pregnant women: Lactobacillus rhamnosus HN001 (6 × 3 109 colony-forming units/d), Bifidobacterium animalis subsp lactis strain HN019 (9 × 3 109 colony-forming units/d) or placebo daily from 35 weeks gestation until 6 months if breast-feeding Infants: same treatment from day 2–16 of life to 2 years	From 35 weeks gestation	Breast feeding mothers: for 6 months Infants: for 2 years since day 2-16 of life	Supplementation with L. rhamnosus HN001 was associated with increased overall glycerol-3 phosphate transport capacity and enrichment of L. rhamnosus. There were no differences in development of eczema by 2 years in either community alpha or beta diversity (*P* > 0.05)	2 years
**(B)** Probiotic given with hydrolyzed/ amino acid-based formulas.								
**References**	**Study**	**Enrolled Patients**	**Hydrolyzed/ amino acid-based formulas+probiotic**	**Probiotic Strain**, **Beginning of Treatment (S)**, **End of Treatment (E)**.	**Pre-natal administration (if yes: duration)**	**Post-natal amministration (if yes: duration)**	**Outcomes**	**Follow-Up (duration)**
Berni Canani et al. (125)	Parallel-arm randomized controlled trial	220 children with cow's milk allergy with a median age of 5.0 months	Probiotic group (*N* = 110): Extensively hydrolyzed casein formula (EHCF) + Lactobacillus rhamnosus GG (LGG) Control group(*N* = 110): Extensively hydrolyzed casein formula (EHCF)	Lactobacillus rhamnosus GG (LGG) S: after randomization E: 3 years	no	36 months	EHCF+LGG reduces the incidence of allergic manifestations (AM)(absolute risk difference was 20.23 (95% CI, 20.36 to 20.10; *P* <.001), and speeds up the time to development of oral tolerance in children with IgE-mediated CMA	36 months

At present, the strains of probiotics tested for prevention of allergy are considered as generally safe during pregnancy and in infancy (81) although adverse effects have not been fully assessed in all studies. We hereby summarize studies and meta-analysis evaluating the efficacy of probiotics on prevention of atopic dermatitis (the most relevant reported outcome) and other allergic manifestations (rhinitis, rhinoconjunctivitis, asthma and/or wheezing, food allergy, and/or their combination).

### Evidence on Prevention of Atopic Dermatitis (AD)

The pioneering study using *Lactobacillus* GG probiotic supplementation in pregnant women, breastfeeding mothers, and infants at high risk of allergy, demonstrated a reduced prevalence of early AD in children compared to the control group (109). Noteworthy, specific Toll-like receptor genetic variations were associated with the protection of eczema by two probiotic strains (*Lactobacillus rhamnosus* HN001 and *Bifidobacterium lactis* HN019), suggesting that individual genetic factors might influence the efficacy and outcome of probiotic supplementation (110).

Table 2 shows details of published RCTs on this topic, with several studies supporting (109, 111–126), while others providing no evidence (127–139), for recommending probiotics in primary prevention of atopic disease.

Conflicting results and conclusions also emerged from reviews, meta-analyses, and guidelines in the last 12 years. Two Cochrane reviews dated 2007 and 2011 did not provide guidance and showed many uncertainties (140, 141). Osborn's first meta-analysis (140) recognized an effect on the prevention of atopic dermatitis, but heterogeneity across studies hampered the draw of definitive conclusions. Afterward, the meta-analysis by Lee et al. (142) analyzed data from a total of 1,581 patients for perinatal administration and showed a preventive effect with a RR of 0.69 (CI: 0.57–0.83). Betsi and colleagues (143) analyzed three studies (584 patients) reporting a significantly decreased incidence of dermatitis in two of them. In 2012 Doege et al. (144), analyzed seven RCTs that reported a modest preventive effect on AD (RR: 0.82, CI: 0.71–0.95; 2,843 patients) with *Lactobacilli*, but not with mixtures of probiotics (128, 129). In the same year, a larger meta-analysis of 13 studies documented a significant preventive effect (RR: 0.79, CI: 0.71–0.88) (145). No difference was found between specific strains nor for target populations (pregnant mothers, breastfeeding mothers, or infants). One year later, a systematic review of 9 studies reported a reduced risk of AD with estimated efficacy ranging from 30 to 70% (146).

In 2015 the WAO (10) reviewed 23 RCTs: in 7 trials the supplementation of probiotics was only in infants (117, 118, 131, 147–150), in 8 trials was in pregnant women and infants [(136, 149, 151–154), while in the other 8 was in pregnant women, breastfeeding mothers and infants (109, 112, 112, 114, 116, 130, 136)]. Fifteen randomized trials of probiotics given to infants measured development of eczema (106, 109, 114, 116–118, 129, 130, 134, 136, 148–150, 155, 156). When used during pregnancy, probiotics were usually supplemented in the last 3 months (10) resulting in a decreased risk of eczema in children, compared to placebo (RR: 0.72, 95%, CI: 0.61–0.85). According to these results, the WAO guideline concluded that (tested) probiotics assumed by pregnant women provide a clear benefit, primarily for the prevention of eczema, in high-risk infants; however, it was a “conditional recommendation,” based on “very low-quality evidence” (10). The same conclusion (conditional recommendation, very low-quality evidence) was drawn in favor of probiotics considering the reduced rate of eczema when compared to placebo (RR 0.61, 95% CI from 0.50 to 0.64) in breastfeeding mothers (10) and in infants (RR 0.81, 95% CI 0.70–0.94).

Two other meta-analyses published in 2015 documented a clear benefit of probiotics only for primary prevention of eczema but did not report significant preventive effects of any other allergic manifestations (108, 151). Zuccotti et al. (151) analyzed 17 studies (4,755 children) and found that probiotics supplementation was associated with a significantly lower relative risk (RR) for developing eczema compared with placebo (RR 0.78; 95% CI: 0.69–0.89), and the most pronounced effect was obtained in particular when heterogeneous mixtures of probiotic strains were used (RR 0.54; 95% CI: 0.43–0.68) (but no with *Lactobacilli* or *Bifidobacteria* alone).

The metanalysis by Cuello-Garcia et al. (108) evaluating 29 studies reported a reduced risk of eczema (follow-up period until 2 years of age) when probiotics were given in the last 3 months of pregnancy (RR 0.71; 95% CI, 0.60–0.84), in breastfeeding mothers (RR 0.57; 95% CI, 0.47–0.69), or both to infants and mothers (RR, 0.80; 95% CI, 0.68–0.94), but not when administered only to infants (RR, 0.83; 95% CI, 0.58–1.19). However, using the GRADE approach, there was a low or very low certainty of evidence due to the “risk of bias, inconsistency and imprecision of results, and indirectness of available research” (108). Results supporting a stronger efficacy of combined perinatal supplementation were reported by two subsequent reviews (34, 153). In particular, according to the Italian review (34), there was “a moderate but constant effect across studies available in the literature for the prevention of atopic dermatitis among children at risk of allergy with the administration of probiotics to the mother during pregnancy and/or after delivery, and to their child during the first 6 months of life.” Similarly, in the most recent review and meta-analysis by Li et al. (153), assessing 28 studies, probiotic supplementation was reported as protective against atopic eczema (OR: 0.69, 95% CI: 0.58–0.82, *P* < 0.0001) and only pre-natal combined with post-natal supplementation obtained a significant reduction. However, it was still open to question when during the gestation the supplementation should start and for how long the intervention should continue in the post-natal period (103, 152).

Moreover, many other clinical studies and meta-analyses reported conflicting results (109, 140, 144, 145, 150, 154–159). These discrepancies could be likely related to different study designs, populations, probiotic strains, and dosages used. As a single strain, LGG showed the most beneficial effect (157) in particular on reducing total and specific immunoglobulin E (IgE) sensitization (158). Conversely, *Lactobacillus acidophilus* has been associated with an increased risk of atopic sensitization (158).

### Evidence on Prevention of Allergic Rhinitis (AR)

Development of allergic rhinitis (AR) in the child following supplementation of probiotics in pregnant women has been evaluated in 5 studies (112, 114, 115, 129, 133) reviewed by Fiocchi et al. (10]. No significant effect has been reported (RR 0.86, 95% CI 0.44–1.7).

Three trials evaluated the onset of AR after supplementing with probiotics breastfeeding mothers and infants (112, 114, 115). Again, relatively few events have been observed and the results were inconclusive (RR 0.86, 95% CI 0.21–3.47). Four trials assessed the development of AR following infant supplementation (120, 128, 138, 142) and confirmed the lack of efficacy of probiotics (RR 0.83, 95% CI from 0.39 to 1.79).

However, in a large cohort study (123), the mothers of 40,614 children were asked to consume two brands of milk and yogurt that contain probiotic strains (*L. acido-philus* LA-5, *B. lactis* Bb12, +/– *L. rhamnosus* GG) during pregnancy. A slight reduction of the risk [adjusted RR (aRR) = 0.87)] of rhinoconjunctivitis at 18–36 months was reported (123). The association between rhinoconjunctivitis and probiotics appeared increased in the case of both the mother (during pregnancy) and the child (from 6 months of age) had consumed these probiotics, as compared when only mother or child consumed.

Conversely, in a longitudinal trial, a higher prevalence of allergic rhino-conjunctivitis at the age of 5–10 years was noted in the probiotic group as compared with the placebo group (36.5 vs. 29.0%, *p* = 0.03) (127).

Therefore, at present, there is no clear evidence that probiotics prevent AR (160), with some reports demonstrating even a detrimental effect (99).

### Evidence on Prevention of Asthma and/or Wheezing

Several systematic reviews and meta-analyses (10, 161, 162) failed to demonstrate a protective effect of probiotics supplementation during pregnancy or early life in the subsequent development of asthma or wheezing. Surprisingly, even an increase in respiratory infections has been reported in children supplemented with probiotics (161).

In 2014 a systematic review and meta-analysis (162) evaluating pre-and post-natal supplementation with probiotics concluded that there was insufficient data to recommend probiotics for the prevention of asthma and wheezing.

In 2015, the WAO analysis (10) of 8 studies (113–115, 129, 133, 135, 136, 163) focusing on the development of asthma/wheezing in the child following administration of probiotics to pregnant women, did not show differences between probiotic and placebo arms (RR 0.93, 95% CI of 0.76–1.1 5). No differences were recorded between probiotic and placebo arms (RR of 1.05, 95% CI from 0.59 to 1.87) in the 4 studies that evaluated asthma/wheezing (113–115, 136) after supplementation of mothers both during pregnancy and during the breastfeeding period and/or supplementation of the infant, Similarly, no differences between the probiotic and placebo groups (RR 0.98, 95% CI from 0.78 to 1.23) were found in the development of asthma/wheezing in the nine studies that evaluate the effect of infants supplementation (10, 113, 114, 117, 118, 129, 131, 133, 136, 163).

### Evidence on Prevention of Food Allergy

A variety of studies provided data that probiotics, including LGG or *L. acidophilus*, do not protect against CMA in infancy (128, 148, 164). Moreover, in a review involving 1,549 infants, Osborn and Sinn (140) stated that the benefit of probiotics in reducing food hypersensitivity is disputable.

In a study conducted by Morisset et al. (150), children at high-risk for the onset of atopic disease were fed with standard infant formula or a fermented infant formula containing heat-killed *Bifidobacterium breve* C50 and *Streptococcus thermophilus* 065. No statistical differences in the incidence of CMA were observed between these two groups, despite infants fed the formula containing probiotics were less sensitized to CMP at skin prick tests (150).

Similarly, a reduced skin prick test sensitivity to CMP or hen's egg protein at age 6 months was reported (165) in children, following supplementation with *Lactobacillus* and *Bifidobacterium* daily to pregnant women (from 36 weeks gestation to delivery) and to infants (from birth through 6 months), when compared to mothers and infants receiving placebo.

However, the results of these studies suggested that probiotics may modulate the development of allergic sensitization to foods, but not necessarily this translates into food allergy prevention (166). Food hypersensitivity is not always associated with symptoms of food allergy, although infants with food sensitization may be more prone to develop a food allergy.

Newborns who received small intestine surgery and antibiotics showed a higher incidence (67%) of CMPI compared to the group supplemented with probiotic treatment (*B. breve)* (119).

Two other studies reported conflicting results with supplementation of LGG (167, 168) (RR 0.88 (95%CI: 0.76–1.03).

Guidelines published in 2014 by the European Academy of Allergy and Clinical Immunology's Taskforce on the prevention of food allergy suggested that there was not enough evidence to support the routine use of probiotics for food allergy prevention (169).

In 2015, the WAO systematic review and meta-analysis (10) reviewed studies evaluating probiotics given to pregnant women (111, 112, 129), breastfeeding mothers (111, 112), and infants (112, 118, 129, 131, 150), and did not document significant effects in reducing the risk of developing food allergy in infants.

Conversely, another meta-analysis (170) in 2016 indicated that probiotics administered pre-natally and post-natally were effective in reducing the risk of atopy and food hypersensitivity (RR 0.77, 95% CI: 0.61–0.98), particularly in families at high risk for allergy. Based on subgroup analyses, the preventive effect was higher when probiotics were administered to both mother and infant, or for a longer duration of the intervention (170), whilst no effect of post-natal probiotic supplementation alone (direct to child) was observed. Only 1 study (135) used solely pre-natal supplementation, and no significant difference in effect was observed between groups. Interestingly, one trial (133) showed that probiotic and prebiotic supplementation during pregnancy and infancy conferred protection preferably to cesarean-delivered children who could not be exposed to remarkable microbial load from a vaginal delivery.

A few studies showed conflicting results of probiotics (LGG) supplementing an extensively hydrolyzed formula in the acquisition of tolerance in infants with CMA (171, 172). Reducing the duration of CMA would be relevant to decrease the possible related risk of other clinical conditions including functional gastrointestinal disorders (173).

To our knowledge, studies exploring the effects of probiotics on confirmed food allergy are surprisingly scant and did not show evidence of benefit compared to non-intervention (111, 128, 165, 170, 174).

### Evidence on Prevention of Whatever Combination of Allergic Diseases Other Than AD

Supplementation with probiotics did not protect against food allergy, asthma, or allergic rhinitis according to two metanalyses published in 2013 (158, 161) and the WAO review (10) that evaluated four randomized trials (106, 131, 136, 156) in 2015 (RR 0.97, 95% CI from 0.85 to 1.12). Two studies evaluating the risk of developing “any allergy” following supplementation in the breastfeeding mother and infant (136, 175) did not report any benefit or harm (RR 1.02, 95% CI 0.71–1.46) (13). The same conclusions were expressed by the other three papers (34, 108, 151). However, Lundelin et al. (176) reported the long-term safety and efficacy of four different strains of probiotics: children receiving LGG perinatally alone or in combination with other strains (*Bifidobacterium lactis* Bb-12, *Lactobacillus paracasei*ST11, and *Bifidobacterium longum*BL999) had a lower risk of developing allergic diseases (allergic rhinitis, eczema, asthma or food allergy) during long-term follow-up (at the age of at least 10 years) compared to the placebo group (47 vs.56%, *p* = 0.09) (176).

A positive effect was also demonstrated in a different RCT (125), involving 220 children (median age of 5 months) with CMA, randomized to either receive extensively hydrolyzed casein formula alone or with L. rhamnosus GG. In the group supplemented with LGG, there was a decrease in the incidence of allergic manifestations (including asthma, eczema, and allergic rhino-conjunctivitis) over a 3-year period and an increased rate of acquisition of tolerance at 36 months (125).

In 2019 a meta-analysis (177) of 17 RCTs (5,264 children) reported there was no significant reduction in the risk of developing asthma after probiotic supplementation compared with controls (RR: 0.86, 95% CI: 0.73–1.01; *p* = 0.06). However, through subgroup analyses, the occurrence of asthma was reduced by L-GG supplementation (RR 0.75; 95% CI: 0.57–0.99; *p* = 0.04) and post-natal only (compared to pre- and post-natal) intervention. The rate of AR, wheeze, and positive aeroallergen SPT results were not different between the two groups. In conclusion, this meta-analysis underlined the importance of specific strain of probiotics and the timing of intervention but also the need for large-sample and high-quality RCTs (177).

Recently, Schmidt et al. (126) examined the effect of supplementation with a mixture of two probiotic strains (*Lactobacillus rhamnosus* and *Bifidobacterium animalis* subsp lactis) in late infancy and early childhood (the mean age at enrollment was 10 months) on the development of allergic diseases and sensitization. As part of the Probicomp Study (138), a double-blind, placebo-controlled intervention trial in which the primary outcome was to reduce infection rate, 290 participants were randomized to either receive a daily mixture of the two probiotic strains (*n* = 144) or placebo (*n* = 146) for 6 months, starting prior to attending daycare. At follow-up (mean age 16.1 months) there was a significantly decreased incidence of eczema in the probiotic group compared to the placebo group (4.2 vs. 11.5%, *P* = 0.036), corresponding to a relative risk of 0.37, but no differences in the incidence of asthma, rhinitis, conjunctivitis, and sensitization across groups were noted (126). However, when the endpoint was grouped as “any allergic disease” (including eczema), 7.6% (*n* = 9) in the probiotic group and 18.9% (*n* = 23) in the placebo group were affected (*P* = 0.010) (126).

Therefore, in conclusion, differences in environmental factors, such as diet or geographic region, in genetic liability as well as in probiotic strains used, timing, and duration of supplementation may be responsible for the heterogeneity in the results of different studies. Overall, diet supplementation with probiotics does not seems to have a beneficial effect in the prevention of allergic manifestations other than AD.

## Synbiotics in the Prevention of Allergic Diseases

First of all, recently it has been revealed that breast milk is not sterile since contains live probiotic *Lactobacillus* (mostly salivarius and fermentum), *Bifidobacterium* species (*B. breve*) (178, 179) as well as *Staphylococcus* and *Streptococcus*. Many factors may influence the composition of breast milk microbiota: the composition of the mother's skin and intestinal microbiota, the mother's health state, and exposure to medications, mostly antibiotics. Moreover, we already discussed the presence and the role in human milk of non-digestible milk human oligosaccharides (HMO) (61). Therefore, we can consider breast milk as a natural synbiotic, containing both probiotics and prebiotics (180) and the beneficial effect of breast milk in the prevention of allergy could be associated with a “synbiotic's effect.”

Regarding supplementation with synbiotics, there are only two RCTs evaluating their role to prevent AD or FA (Table 3A). The first study (156) reported a reduction in the rate of eczema and IgE-associated allergic diseases, including challenge-proven FA, by synbiotic supplementation. The second study documented a reduced eczema risk with synbiotic supplementation but did not study FA (181). However, a meta-analysis of these studies labeled these results as not significant, underling the wide CI (186).

**Table 3:** Synbiotics administration in prevention of allergic disorders.

**(A)** Synbiotic + Standard formula/breastfeeding.									
**References**	**Study**	**Enrolled Patients**	**Synbiotic** **+** **Standard formula/ breast feeding**	**Prebiotic substance**, **Beginning of Treatment (S), End of Treatment (E)**.	**Probiotic Strain, Beginning of Treatment (S), End of Treatment (E)**.	**Pre-natal administration (duration)**	**Post-natal administration (duration)**	**Outcomes**	**Follow-Up (duration)**
Kukkonen et al. (156)	double-blind randomized, placebo controlled trial	− 1223 pregnant woman carrying high- risk children and their infants: - *N* = 461 mothers-infants received symbiotic - *N* = 464 mothers-infants received placebo	- Synbiotics group: - mothers: 1 capsule containing 4 probotics twice daily - infants received 1 opened capsule containing the same probiotics mixed with galacto-oligosaccharides once daily Placebo group: capsules containing microcrystalline cellulose, and the infants received syrup without galacto-oligosaccharides	Synbiotics group: infants received 1 opened capsule containing the same probiotics mixed with drops of sugar syrup containing 0.8 g of galacto-oligosaccharides once daily S(women): 2–4 weeks before delivery E (Women): at delivery S (infants): birth E (infants): 6 months	1 capsule containing L. rhamnosus GG(ATCC 53103), 5 × 10^9^ cfu; L. LC705 (DSM 7061), 5 × 10^9^ cfu; B. breve Bb99(DSM 13692), 2 × 10^8^ cfu; and P. freudenreichii ssp. shermanii JS(DSM 7076), 2 × 10^9^ cfu, twice daily S(women): 2–4 weeks before delivery E (Women): at delivery S (infants): birth E (infants): 6 months	2–4 weeks before delivery	For 6 months	There was no effect of probiotic supplementation compared with placebo on the cumulative incidence of any allergic disease (OR, 0.85; 95% CI, 0.64–1.12). There was a reduced occurrence of Eczema in the probiotic group (OR, 0.74; 95% CI, 0.55–0.98)	2 years
Roze et al. (181)	double-blind, randomized, multicenter trial	Ninety-seven non-brestfed term neonates:	Symbiotics group (n 48): Standard formula + symbiotics Control group(n 49): standard formula	experimental formula containing the two strains of probiotics +96% galacto-oligosaccharides and 4 % short-chain fructo-oligosaccharides	experimental formula containing L. rhamnosus LCS- 742(1.4 × 10^8^), B. longum subsp infantis M63 (1.4 × 10^8^) and prebiotics:	no	For 6 months	Atopic dermatitis was less frequently observed in the experimental group (2.6% vs. 17.8%, *P* < 0.05)	6 months
**(B)** Synbiotic +Hydrolyzed/ amino acid-based formulas.									
**Refereences**	**Study**	**Enrolled patients**	**Hydrolyzed/ amino acid-based formulas+synbiotic**	**Prebiotic substance**, **Beginning of treatment (S), End of treatment (E)**.	**Probiotic strain, dose Beginning of treatment (S), End of treatment (E)**.	**Pre-natal administra-tion (duration)**	**Post-natal administration (duration)**	**Outcomes**	**Follow-up (duration)**
van der et al. (182)	double-blind, placebo-controlled multicentre trial	ninety full-term infants, aged <7 months with AD	Synbiotic group: extensively hydrolyzed whey-based formula with additional synbiotics [B. breve M-16V and a galacto/fructooligosaccharide mixture] Control group: same formula without synbiotics	mixture of 90% scGOS and 10% lcFOS 0.8 g/100 ml S: <7 month E: after 12 weeks	B. breve M-16V (1.3 × 10^9^ cfu/100 ml) S: <7 month E: after 12 weeks	no	12 weeks	The SCORAD score improvement (AD severity) did not differ between the synbiotic and the placebo group. In the synbiotic group there was a significantly higher percentage of bifidobacteria (54.7% vs. 30.1%, *P* < 0.001) and significantly lower percentages of *Clostridium lituseburense* /*Clostridium histolyticum* (0.5 vs. 1.8, *P* = 0.02) and *Eubacterium rectale* /*Clostridium coccoides* (7.5 vs. 38.1, *P* < 0.001) after intervention than the placebo group	12 weeks
van der et al. (183)	double-blind, placebo-controlled multicentre trial	ninety full-term infants, aged <7 months with AD	Synbiotic group: extensively hydrolyzed whey-based formula with additional synbiotics [B. breve M-16V and a galacto/fructooligosaccharide mixture] Control group: same formula without synbiotics	mixture of 90% scGOS and 10% lcFOS 0.8 g/100 ml S: <7 month E: after 12 weeks	B. breve M-16V (1.3 × 10^9^ cfu/100 ml) S: <7 month E: after 12 weeks	no	12 weeks	infants in the synbiotics group have a lower prevalence of asthma-like symptoms (frequent wheezing) and asthma medication use at 1-year follow-up than those who received placebo [13.9% vs. 34.2%, absolute risk reduction (ARR)]20.3%, 95% CI −39.2% to −1.5% and (5.6% vs. 25.6%, ARR−20.1%, 95% CI −35.7% to −4.5%)	1 years
Candy et al. (184)	multicenter, double-blind, randomized controlled trial	Term infants <13 months old, with suspected non-IgE-mediated CMA	Symbiotic group (*N* = 35): amino-acid-based formula (AAF) contained a prebiotic blend and a probiotic strain control group (*N* = 36): commercially available AAF	chicory-derived neutral oligofructose, long-chain inulin (9:1 ratio at a total concentration of 0.63 g/100 ml S: <13 months E: after 8 weeks	*Bifidobacterium breve* M-16V) at a concentration of 1.47 × 10^9^ CFU/100 mL S: <13 months E: after 8 weeks	no	8 weeks	There was a significantly higher median percentage of *Bifidobacteria* w (*p* < 0.001) in the test group than in the control subjects (35.4% vs. 9.7%), whereas a lower percentage of *Eubacterium* rectale/*Clostridium coccoides* group in feces (9.5% vs. 24.2%) and similar to that detected in breastfed infants (55% and 6.5%, respectively). There was no statistically significant changes over 8 weeks in the reported scores for skin symptoms. SCORAD decreased between weeks 0 and 8, from 12.83 ± 18.84 to 9.63 ± 12.45 in the test group and from 14.43 ± 19.74 to 7.06 ± 10.01 in the control group	8 weeks
Fox et al. (185)	double-blind, randomized, controlled multicenter trial	Term infants <13 months old, with suspected non-IgE-mediated CMA	Symbiotic group (*N* = 35): amino-acid-based formula (AAF) contained a prebiotic blend and a probiotic strain control group(*N* = 36): commercially available AAF Healthy reference group (*N* = 51)	chicory-derived neutral oligofructose, long-chain inulin (9:1 ratio at a total concentration of 0.63 g/100 ml) S: <13 month E: after 8 weeks	*Bifidobacterium breve* M-16V at a concentration of 1.47 × 10^9^ CFU/100 mL S: <13 month E: after 8 weeks	no	8 weeks	The supplementation of AAF with specific synbiotics induced a sustained improvement in gut microbiota composition. The median percentages of bifidobacteria were significantly higher at week 26 in the test group than controls [47.0% vs. 11.8% (*p* < 0.001)], whereas percentages of *ER/CC* were significantly lower [(13.7% vs. 23.6% (*p* = 0.003)]. The use of dermatological medication and reported ear infections were lower in test vs. control, *p* = 0.019 and 0.011, respectively	26 weeks

Other studies evaluating synbiotic supplementation (182, 184, 185) documented only fecal microbiota changes. Van der Aa et al. (182) reported the effects of a mixture of synbiotics, *B. breve* M-16V, and scGOS/lcFOS, added to an extensively hydrolyzed formula and administered for 3 months to formula-fed infants, in resembling the metabolic profile of breast-fed infants, by modulating the composition and the metabolic activity of gut microbiota. The same synbiotic mixture significantly reduced the prevalence of asthma-like symptoms and of asthma medications use at 1-year follow-up (183) (Table 3B). A recent multicenter double-blind RCT (184) documented the effects of an amino acid-based formula (AAF) supplemented with *B. breve* M-16V and fructo-oligosaccharides, in 35 infants with suspected non-IgE-mediated CMA, compared to 36 controls: after 8 weeks of administration, there was a significantly lower median percentage of Bifidobacteria in the control group (9.7 vs. 35.4%), whereas *Eubacterium rectale*/*Clostridium coccoides* group in feces was lower in the synbiotic group (9.5 vs. 24.2%) and similar to breastfed infants (55 and 6.5%, respectively). A subsequent trial with the same study groups and design and formulas confirmed the same changes in the fecal microbiota at 26 weeks (185).

Overall, the limited available data on the role of supplementation with synbiotics for the prevention of allergic diseases cannot allow a definitive conclusion.

## Discussion

Even if many studies, reviews, and meta-analyses, and several guidelines are available on this topic, the overall preventive effect of prebiotic/probiotic supplementation on allergic diseases remains unclear. The safety profile of these agents is excellent without significant adverse events in any revised literature. Regarding probiotics, the combined strategy of pre-natal and post-natal supplementation has been demonstrated promising in preventing atopic eczema; however, question when during the gestation and for how long the intervention should continue in the post-natal period is still open. There is no clear evidence that probiotics have a beneficial effect on the development of AR, asthma, and/or wheezing. Thus, routine use of probiotics for preventive purposes cannot be recommended. Future studies focusing on the primary prevention of allergic diseases should investigate the optimal strains, dosing, duration of therapy, and longer follow-up times are warranted (170).

Currently, most guidelines, including those from the European Academy of Allergy and Clinical Immunology (169), and the European Society for Pediatric Gastroenterology, Hepatology, and Nutrition (98), due to lack of definitive evidence, do not recommend probiotic supplementation to prevent allergic diseases. Conversely, the World Allergy Organization (WAO), recommends probiotics for high-risk infants, for the potential benefits, in pregnant women, in breastfeeding women, and in infants, in preventing AD (10). However, the WAO did not consider specific strains and concluded that available data are not enough to support intervention for preventing any form of allergic disease by the routine use of probiotics, except for infants at high risk for eczema (10). Indeed, the activity of the probiotics is believed to depend on the bacterial strain type, on the dose, and on the intervention timing. Probiotics have been administered during pregnancy, lactation, to neonates, or later in infancy with different results, and without clear data on the right timing. Furthermore, careful selection of treatment strategy during pregnancy and early infancy is mandatory to identify the best target population, to achieve positive and limit negative outcomes.

The existing evidence on prebiotics is even more limited. The metanalyses and systematic reviews about prebiotics and prevention of allergic diseases concluded that the existing evidence is insufficient to draw conclusions (96, 98) on their preventive effect due to the high heterogeneity of the various studies. The WAO recommends supplementation with prebiotics in infants that are not exclusively breastfed, even if there is a very low quality of evidence (99). Cuello-Garcia et al. (100) showed a potential activity of prebiotic supplementation in infants resulting in asthma or wheezing risk reduction. However, the evidence is very low. The activity of prebiotics on the prevention of atopic eczema is observed, but data are inconclusive. Therefore, the authors (100) stated that available data on supplementation with prebiotics in terms of allergy risk reduction is not so strong to support or reject the concept of benefit or harm with prebiotic.

In addition, after a careful review of the available literature according to the method of administration (Tables 1–3), we noticed that the positive outcomes in prevention were reported more frequently among the group of studies in which prebiotics or probiotics were given with hydrolyzed/amino acid based formula, compared to those in which were administered alone or with a standard formula, suggesting a possible synergic effect with a hypoallergenic formula that needs to be confirmed with further studies. Moreover, due to the known bifidogenic effect of lactose, its content in different formulas (especially hydrolyzed/aminoacid based formulas) should be taking into account when evaluating the results of prebiotic studies, as might be a possible confounding factor. In addition, it has been recently suggested the possible role of different approaches to complementary feeding in the development of the gut microbiota in early life (87, 187, 188). Therefore, the type of the first solid foods introduced could play a relevant role in shaping the infant's gut microbiota, as well as different intakes of foods naturally containing prebiotic components, all acting as possible confounding factors when evaluating results of pre/probiotic studies.

In conclusion, further RCTs in populations with high or low atopy risk, taking into account a possible synergic effect with other factors, are needed to carefully define the effectiveness of prebiotics/probiotics by itself for allergy prevention. At this time, on the basis of currently available data, supplementation with probiotics for prevention of allergies in children cannot be recommended, even if it is possible to underline the net benefit in high-risk infants in the prevention of eczema, as this effect is predominantly constant across studies available in the literature. However, the optimal strains, dose and timing, and duration of supplementation are still unknown, although a combined pre- and post-natal intervention appeared of stronger benefit. Moreover, the evidence for recommendation of prebiotic supplementation in infants who are not exclusively breastfed is of very low certainty and quality. Therefore, conclusive evidence is still lacking to be able to recommend routine use of pre/probiotics for allergic preventive purposes.

## Author Contributions

SSe, ED'A, and LP contributed to conception and design of the review, interpretation of data, drafting the article, and final approval of the version to be published. MB, SSa, VT, ES, FT, and DC contributed to interpretation of data, drafting the article, and final approval of the version to be published. All authors contributed to the article and approved the submitted version.

## Conflict of Interest

The authors declare that the research was conducted in the absence of any commercial or financial relationships that could be construed as a potential conflict of interest.
